# Identification of the vascular endothelial growth factor signalling pathway by quantitative proteomic analysis of rat condylar cartilage

**DOI:** 10.1002/2211-5463.12155

**Published:** 2016-12-20

**Authors:** Liting Jiang, Yinyin Xie, Li Wei, Qi Zhou, Xing Shen, Xinquan Jiang, Yiming Gao

**Affiliations:** ^1^ Department of StomatologyRuijin Hospital Affiliated to Shanghai Jiao Tong University, School of MedicineChina; ^2^ Department of ProsthodonticsShanghai Key Laboratory of StomatologyNinth People's Hospital Affiliated to Shanghai Jiao Tong University, School of MedicineChina; ^3^ State Key Laboratory of Medical GenomicsShanghai Institute of HematologyRuijin Hospital Affiliated to Shanghai Jiao Tong University, School of MedicineChina; ^4^ Shanghai Institute of Traumatology and OrthopaedicsRuijin Hospital Affiliated to Shanghai Jiao Tong University, School of MedicineChina

**Keywords:** condylar cartilage, isobaric tags for relative and absolute quantification, mechanical loading, proteomic analysis, vascular endothelial growth factor

## Abstract

Angiogenesis mediated by vascular endothelial growth factor (VEGF) is known to play an important role in regulating cartilage remodelling and endochondral ossification. However, the details of how VEGF signalling mechanisms affect condyle remodelling in response to alterations in functional loading remains unclear. To explore this, eighty 16‐day‐old male SD rats were divided into two equal groups which were fed either a soft/powdery diet or a hard diet for 4 weeks; the stiffness of the diet results in alteration of mastication force and hence temporomandibular joint (TMJ) development. We performed a proteomic analysis of rat condylar cartilage using isobaric tags for relative and absolute quantification (iTRAQ) labelling, followed by 2D nano‐high performance liquid chromatography and MALDI‐TOF/time‐of‐flight technology. After protein identification, we used biological information analysis to identify the differentially expressed proteins associated with the VEGF signalling pathway. Among the identified differentially expressed proteins, we found VEGF signalling mainly *via* the p44/42 MAPK and p38 mitogen‐activated protein kinase (MAPK) pathways in condylar cartilage, including VEGFD, VGFR2, KPCB, KPCT, KPCZ, ARAF, RASN, PLCG2, PLCG1, JUN and M3K12. Furthermore, four representative protein candidates, VEGF, p38 MAPK and p44/42 MAPK/phospho‐p44/42 MAPK, were confirmed by immunohistochemical staining and western blot. Our data suggest that VEGF might play an important role in TMJ development and remodelling in response to alterations in functional loading through the p44/42 MAPK and p38 MAPK signalling pathway. This study provides new clues to the understanding of the signalling mechanism responsible for VEGF production in response to different masticatory functions at the protein level.

**Abbreviations**
H&E: haematoxylin and eosin
HPLC: high‐performance liquid chromatography
IHC: immunohistochemical
iTRAQ: isobaric tags for relative and absolute quantitation
MALDI‐TOF/TOF: matrix‐assisted laser desorption ionization time‐of‐flight/time‐of‐flight
MCC: mandibular condylar cartilage
MS: mass spectrometry
RP: reverse‐phase
SCX: strong cation exchange
SDS: sodium dodecyl sulphate
TMJ: temporomandibular joint
VEGFR: VEGF receptor
VEGF: vascular endothelial growth factor

Mastication provides a crucial mechanical stimulus for jawbone remodelling. Reduced masticatory loading induced by a soft diet negatively affects the jaw muscle activity and the masticatory force and strength 1. Many previous studies have shown that masticatory loading directly positively influences jaw muscle fibres 2, 3 and mandibular morphology, mineral density and temporomandibular joint (TMJ) strength 4 in growing 5 and adult animals 6. The cells and microstructure of the mandibular condyle are particularly responsive to biomechanical stress, such as that of mastication 7, 8.

The invasion of new vasculature into cartilage is the first vital step in the process of endochondral ossification on the mandibular condyle. The mandibular condylar cartilage (MCC) itself is an alymphatic and nonvascular tissue. Angiogenesis brings in circulating factors that promote the replacement of cartilage by bone growth and remodelling, leading to endochondral bone formation 9. Vascular endothelial growth factor (VEGF) is the single most important mediator regulating vascular development and angiogenesis. It is thought to be synthesized by hypertrophic chondrocytes in the epiphyseal growth plate and is essential for extracellular matrix remodelling, angiogenesis and endochondral ossification 10. Recent evidence supports this notion because VEGF could be found in growing MCC but not new‐born MCC 11, 12. Numerous studies have shown that VEGF is central to promoting endochondral bone formation by affecting the proliferation and migration of endothelial cells 13 and chondrocytes 14 or inducing neovascularization in response to physiological/nonphysiological mechanical load in MCC 15. However, the details of VEGF signalling mechanisms in the condyle need further investigation.

Isobaric tags for relative and absolute quantitation (iTRAQ) technology is a powerful and popular proteomic labelling method used in the search for markers or molecular mechanisms in health or disease conditions, as it can display thousands of proteins simultaneously 16, 17. In the current study, we used iTRAQ analysis to determine changes in the VEGF signalling pathway in MCC after mastication. Rodents were fed a soft versus hard diet to reproduce the reduction of masticatory function experimentally. After protein identification, we focused on the analysis of biological information to screen out the differentially expressed proteins that are associated with the VEGF signalling pathway. In the second step, we selected four representative proteins [VEGF, p38 mitogen‐activated protein kinase (MAPK) and p44/42 MAPK/phospho‐p44/42 MAPK proteins] to validate the results of proteomic analysis by immunohistochemical (IHC) and western blot.

## Materials and methods

### Experimental model and tissue preparations

Sixteen‐day‐old male Sprague–Dawley rats without mastication were used in this study. All procedures were approved by the Ethics Committee for Animal Care and Use of the Research Center for Experimental Medicine of Ruijin Hospital. Eighty rats (40 in each group) were divided into two groups: those fed a soft/powdery diet versus those fed a hard diet as described previously 18. Similar quantities of water and food were offered to both groups *ad libitum* daily. The animals were sacrificed 4 weeks later.

### Preparation of condylar cartilage proteins and proteomic analysis

**Figure 1:**
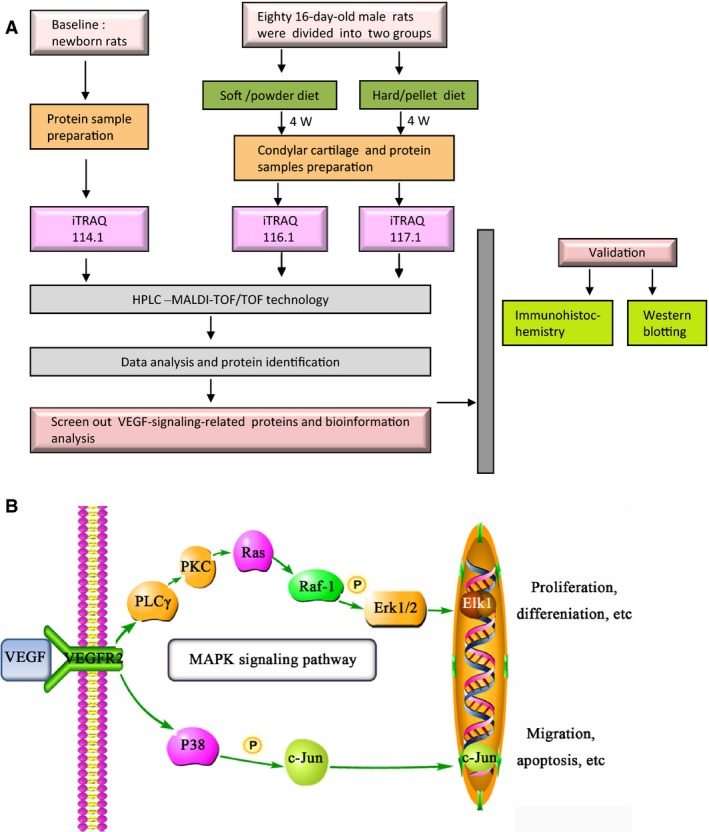
(A) The experimental design for iTRAQ proteomic analysis. (B) Illustration of VEGF signalling pathway in condylar cartilage according to the proteomic results.

Forty condylar cartilages per group were mixed together, frozen in liquid nitrogen, pulverized mechanically and suspended in RIPA lysis buffer (Beyotime, Shanghai, China). The lysed samples were vigorously vortexed for 30 min on ice and centrifuged for 15 min at 14 000 ***g*** at 4 °C. Then, protein concentrations were estimated by BCA assay (Beyotime). After being reduced, alkylated, and digested with trypsin (Promega, Madison, WI, USA) at 37 °C overnight, 100 μg protein per group was labelled with 4‐plex iTRAQ reagents according to the standard manufacturer's supplied protocol (iTRAQ Reagent Multi‐plex Kit; AB Sciex, Framingham, MA, USA; schematics of the experimental design are shown in Fig. 1A). Peptides from the baseline (new‐born rat condyles), soft diet group and hard diet group were labelled with 114.1, 116.1 and 117.1 respectively.

The samples were then separated by two‐dimensional separation using 2D nano‐high‐performance liquid chromatography (HPLC; LC‐20A; SHIMADZU, Tokyo, Japan), including the first dimension using a strong cation exchange (SCX) column and the second dimension using a reverse‐phase (RP) analytical capillary column (Thermo, Waltham, MA, USA). Briefly, the mixed iTRAQ‐labelled peptides were first diluted in SCX buffer A (10 mm ammonium formate in 0.1% v/v formic acid), followed by a gradient elution of 0%, 20%, 50% and 100% SCX buffer B (500 mm ammonium formate in 0.1% v/v formic acid) over 24 min (at 6‐min intervals per gradient). The SCX fractions were then separated by an RP analytical capillary column using a time‐linear gradient of buffer A (0.1 v/v trifluoroacetic acid in 5% v/v acetonitrile) to buffer B (0.1 v/v trifluoroacetic acid in 90% v/v acetonitrile). The column flow rate was maintained at 2 μL·min^−1^ of CHCA matrix solution over 40 min. Positive ions were then spotted onto target plates for MALDI‐TOF/TOF −4700 mass spectrometry (MS) measurements with the AccuSpot system (SHIMADZU). The MALDI‐TOF spectra were acquired in TOF/TOF mode using 10 laser shots per spectrum, while TOF/TOF fragmentation spectra were acquired using 20 laser shots per fragmentation spectrum. The scan range was 800–4000 *m/z* and the frequency was 20 scans per time. Five precursor ions per well were selected from four plates (192 wells per plate) for tandem MS identification, and approximately 3000 precursor ions were selected for the target plates. The parameters for MS analysis were a signal‐to‐noise threshold of 20 and a minimum area of 100 and a resolution higher than 10 000 with a mass accuracy of 20 p.p.m. The following mass search parameters were set: mass spectra over the *m/z* range of 800–4000 Da; MS tolerance ±0.15 *m/z* and MS/MS tolerance ±0.1 *m/z*, and an allowance of missed cleavage of 1, with consideration for variable modifications. The results were gathered by database searching against mascot software (version 2.1; Matrix Science, London, UK) and the rat SWISS‐PROT protein database (Release 2014_01). The detected protein threshold was set to 80% confidence. All spectra used for protein ratio calculations were unique to the given proteins. Cut‐off confidence values accepting protein identification for Mascot was 80%. Only proteins detected in every biological replicate were included, and they must have contained at least two unique high‐scoring peptides. iTRAQ ratios were analysed automatically by gps explorer(tm) v3.6 software (AB Sciex). The iTRAQ ratio of increased proteins was greater than 1.20, and the iTRAQ ratio of decreased proteins was < 0.80 19. For bioinformatics analysis, cluster analysis was performed according to their cellular component, biological process and molecular function. The PANTHER pathway database (http://www.pantherdb.org/) and the web‐based david software (https://david.ncifcrf.gov/) were used to analyse and annotate the functions of many proteins.

### Haematoxylin and eosin staining and immunohistochemical staining

**Figure 2:**
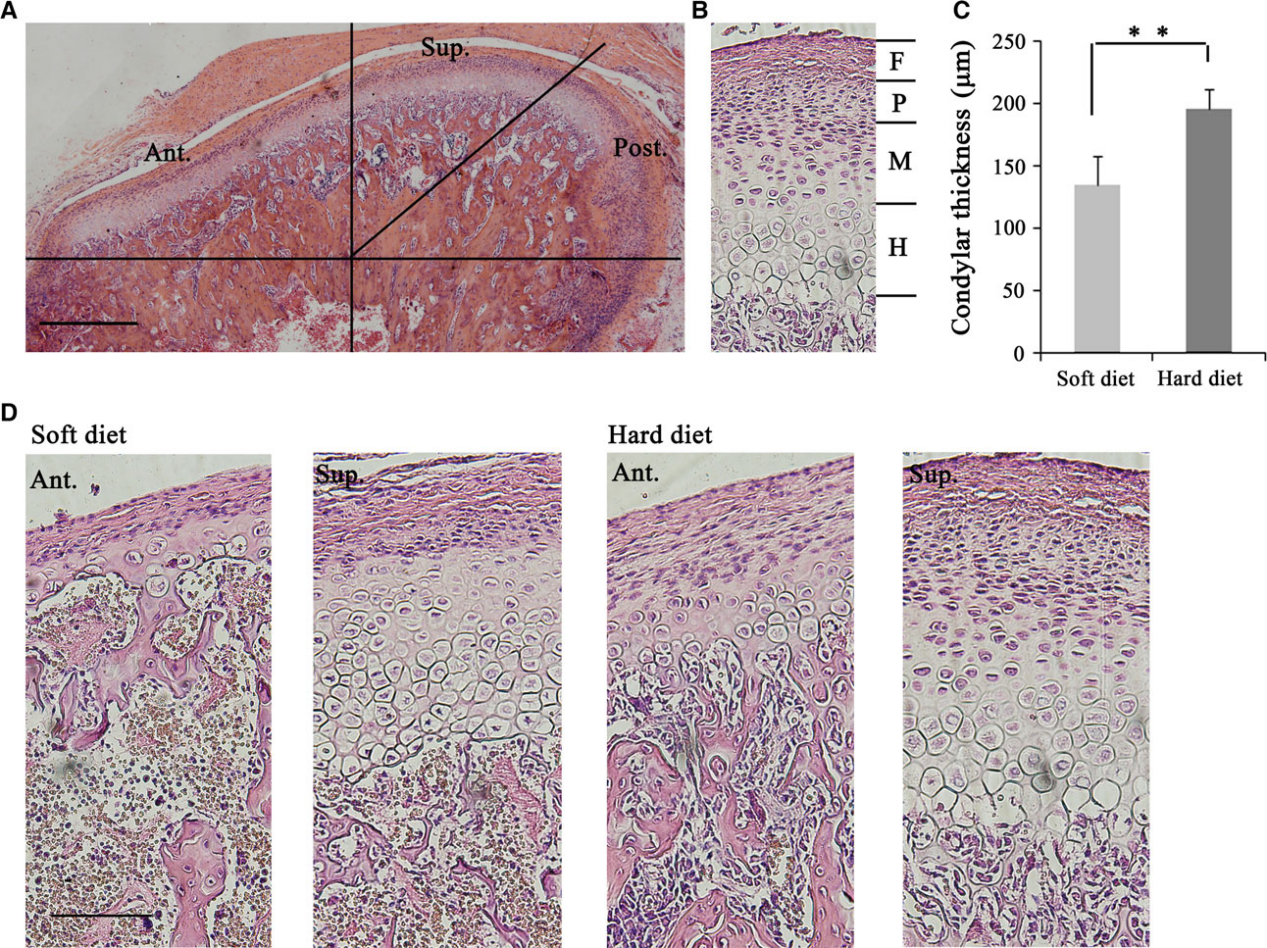
Histological examination of condylar cartilage from rats fed soft and hard diet. (A) Representative image showing sagittal section of condylar cartilage stained with H&E and illustrated the anterior, central and posterior region of condylar cartilage (original magnification 4×, Bar = 500 μm). (B) Representative images showing that condyle cartilage consisted of subchondral bone (S) and cartilage, which included the fibrous layer (F), proliferative layer (P), the maturing layer (M) and hypertrophic layer (H). (C) Comparison of the cartilage thickness of anterior region between soft diet and hard diet group. (D) Representative images showing superior and anterior region of condylar cartilage of rats fed soft and hard diet (original magnification 20×, Bar = 100 μm). **P < 0.01.

Immediately after sacrifice, 10 condylar samples in each group were embedded for histological analysis. The condyles were fixed in 4% paraformaldehyde for at least 48 h and then decalcified in ethylene diamine tetra‐acetic acid solution at 4 °C for 4 weeks. The samples were paraffin‐embedded and 5‐μm sagittal sections were obtained. After haematoxylin and eosin (H&E) staining, the condylar cartilage could be stratified into four layers, the fibrous layer, proliferative layer, the maturing layer and hypertrophic layer, and divided into three regions: anterior, superior and posterior (Fig. 2A,B). As the most drastic changes were observed in the anterior region of the condyle, the thickness of the anterior region was measured in a blinded, nonbiased manner by image analysis software (image‐pro plus 6.0; Media Cybernetics, Silver Spring, MD, USA).

A standard IHC method was used according to the manufacturer's recommended protocol. Endogenous peroxide was quenched or destroyed by incubating it with 3% hydrogen peroxide for 20 min at room temperature. The sections were then reacted with the following primary antibodies (proteins were selected according to the results of proteomic analysis) at 4 °C overnight respectively: anti‐VEGF antibody (1 : 200, ab46154; Abcam, Cambridge, UK), p38 MAPK antibody (1 : 100, #9212; Cell Signaling, Beverly, MA USA), p44/42 MAPK antibody (1 : 200, #4686; Cell Signaling) and phospho‐p44/42 MAPK (Erk1/2) antibody (1 : 200, #4370; Cell Signaling). As negative controls, the primary antibodies were substituted with phosphate‐buffered saline (PBS). The slides were subsequently washed with PBS twice and then incubated with biotinylated secondary antibody for 30 min at room temperature, washed again and finally incubated with streptavidin–peroxidase complex for 15 min at room temperature. The antibody staining was performed with peroxidase/diaminobenzidine (DAB; Sigma Chemical Co., St. Louis, MO, USA) and the slides were counterstained in the nucleus with haematoxylin for 15 s. The localization of IHC‐positive cells was examined microscopically and semiquantitative analysis was performed. The integrated optical density of condylar IHC staining was measured and statistics analysed in three same anatomical area per section (mid‐sagittal).

### Western blot analysis

To confirm the results of proteomic analysis, four representative proteins, VEGF, p38 MAPK and p44/42 MAPK/phospho‐p44/42 MAPK, were validated by western blot. According to standard procedures, 30 condylar cartilages per group were mixed together and 200 μL RIPA lysis buffer (Beyotime), 2 μL PMSF (Sigma) and 2 μL phosphatase inhibitor cocktail (Roche, Applied Science, Mannheim, Germany), frozen in liquid nitrogen and ground, were added. The lysates were spun down at 14 000 ***g*** for 10 min at 4 °C and the protein concentration was evaluated by Bradford protein assay (Bio‐Rad Laboratories Inc., Hercules, CA, USA). The protein extracts were loaded and separated on 10% SDS/PAGE, and then transferred to a polyvinylidene fluoride (PVDF) membrane (Millipore, Bedford, MA, USA). Transferred membranes were incubated in blocking solution with TBST buffer containing 5% w/v nonfat milk and incubated with primary antibodies as follows: anti‐GAPDH antibody (1 : 1000, ab8245; Abcam), anti‐VEGF antibody (1 : 500, ab46154; Abcam), p38 MAPK antibody (1 : 1000, #9212; Cell Signaling), p44/42 MAPK antibody (1 : 1000, #4686; Cell Signaling) and phospho‐p44/42 MAPK (Erk1/2) antibody (1 : 1000, #4370; Cell Signaling) overnight in TBST (10 mm Tris/HCL, pH 7.5, 150 mm NaCl, 0.1% Tween‐20) supplemented with 1% BSA at 4 °C. After hybridization with corresponding secondary antibodies from Cell Signaling, the membrane was visualized using an ECL western blotting detection system (ECL kits, #170‐5060; Bio‐Rad Laboratories Inc.). The results were digitized using a GE Image Quant LAS 4000 mini analyser (GE, Marlborough, MA, USA). The relative abundance of four proteins was analysed by obtaining the ratio of the normalized densitometric values between the soft and hard diet groups.

### Statistical analysis

All statistical data (including histological and western blot data) were expressed as the mean ± standard deviation (SD) using spss version 13.0 (SPSS Inc., Chicago, IL, USA) and comparisons were analysed by one‐way ANOVA followed by *t*‐test. Differences were considered significant at a *P*‐value < 0.05.

## Results

### iTRAQ proteomic analysis identified that VEGF signalling increased after mastication

We performed iTRAQ analysis coupled with 2D nano‐HPLC and MALDI‐TOF/TOF technology and identified that 805 proteins were differentially expressed between the two groups. Only proteins that were identified as at least two unique high‐scoring peptides were detected. To functionally verify some intriguing proteins identified by iTRAQ, we utilized a web‐based tool, david, to highlight the MAPK signalling pathway that participated in MCC mechanotransduction. According to the GO and PANTHER databases, we screened out 10 VEGF signalling‐related proteins, M3K12 and JUN in condylar cartilage in response to different types of functional loading, and eight of them displayed an up‐regulated trend. Additionally, most proteins, including VEGFR2, PLCG1, PLCG2, KPCB, KPCT and RASN, were involved in the MAPK signalling pathway, and M3K12 and JUN were the key signalling molecules of the P38 MAPK pathway (shown in Table 1 and Table S1). The pathway information generated by KEGG successfully recognized the classical MAPK pathway and P38 MAPK pathway involved in this process. The VEGF (encoded by the *VEGFD* gene) and p38MAPK (encoded by the *M3K12* gene) proteins were up‐regulated in the hard diet group when compared to the soft diet group. Moreover, as MAPKs can be inactivated by a cascade of dephosphorylation and activated by phosphorylation, and several upstream (such as PKC, Ras) and downstream (such as cPLA2, MKP, encoded by the *DUS6* gene) targets of ERK1/2 were identified in our proteomic results. Thus, we selected the VEGF, p44/42 MAPK (Erk1/2), p‐p44/42 MAPK (Erk1/2) and P38 MAPK proteins for further verification (the VEGF signalling pathway is shown in Fig. 1B.).

**Table 1:** List of VEGF signalling‐related proteins by iTRAQ proteomic analysis from the condylar cartilage of rats fed 4 weeks of soft and hard food diet

Gene ID	Protein name	KEGG pathway	116.1/114.1	117.1/114.1
VEGFD	Vascular endothelial growth factor D precursor	rno04060:Cytokine–cytokine receptor interaction; rno04150:mTOR signalling pathway, rno04510:Focal adhesion	0.44	0.54
VGFR2	Vascular endothelial growth factor receptor 2 precursor	rno04060:Cytokine–cytokine receptor interaction; rno04144:Endocytosis	0.63	0.63
KPCB	Protein kinase C beta type	rno04010:MAPK signalling pathway; rno04012:ErbB signalling pathway; rno04020:Calcium signalling pathway	0.60	0.51
ARAF	A‐Raf proto‐oncogene serine/threonine‐protein kinase		1.78	1.66
RASN	GTPase NRas precursor	rno04010:MAPK signalling pathway; rno04012:ErbB signalling pathway	2.39	2.96
NOS3	Nitric‐oxide synthase, endothelia	rno00330:Arginine and proline metabolism; rno04020:Calcium signalling pathway	0.40	0.58
PLCG2	Phospholipase C‐gamma‐2	no00562:Inositol phosphate metabolism; rno04012:ErbB signalling pathway, rno04020:Calcium signalling	0.70	0.74
KPCT	Protein kinase C theta type	rno04270:Vascular smooth muscle contraction; rno04530:Tight junction; rno04920:Adipocytokine signalling pathway	0.61	0.64
NOS1	Nitric‐oxide synthase	rno00330:Arginine and proline metabolism; rno04020:Calcium signalling pathway	0.88	0.86
PLCG1	Phospholipase C‐gamma‐1	rno00562:Inositol phosphate metabolism; rno04012:ErbB signalling pathway; rno04020:Calcium signalling	0.87	1.01
KPCZ	Protein kinase C zeta typ	rno04144:Endocytosis; rno04530:Tight junction; rno04910:Insulin signalling pathway	0.57	0.77

### Mastication increases condylar cartilage thickness and VEGF immunostaining

To assess tissue‐level changes in condylar cartilage after 4 weeks of hard mastication, we examined the cartilage by H&E staining (Fig. 2). Figure 2A shows the anterior, superior and posterior regions of the condylar cartilage. The anterior region is in more contact with food, is directly involved in the mastication process and directly experiences mechanical forces. Figure 2B shows that the condylar cartilage can be divided into four layers: the fibrous layer, proliferative layer, maturing layer and hypertrophic layer. Cells in the proliferative layer have the ability to differentiate into chondrocytes, and their differentiation pathway is thought to be regulated by biomechanical force. The activity of collagen synthesis is high in the maturing layer, while the hypertrophic layer is required for condylar endochondral ossification. The anterior cartilage thickness was markedly increased in the hard diet group compared to the soft diet group (Fig. 2D). Quantification of the cartilage thicknesses showed that the anterior cartilage thickness was indeed significantly increased in the hard food group compared to the soft diet group (Fig. 2C).

**Figure 3:**
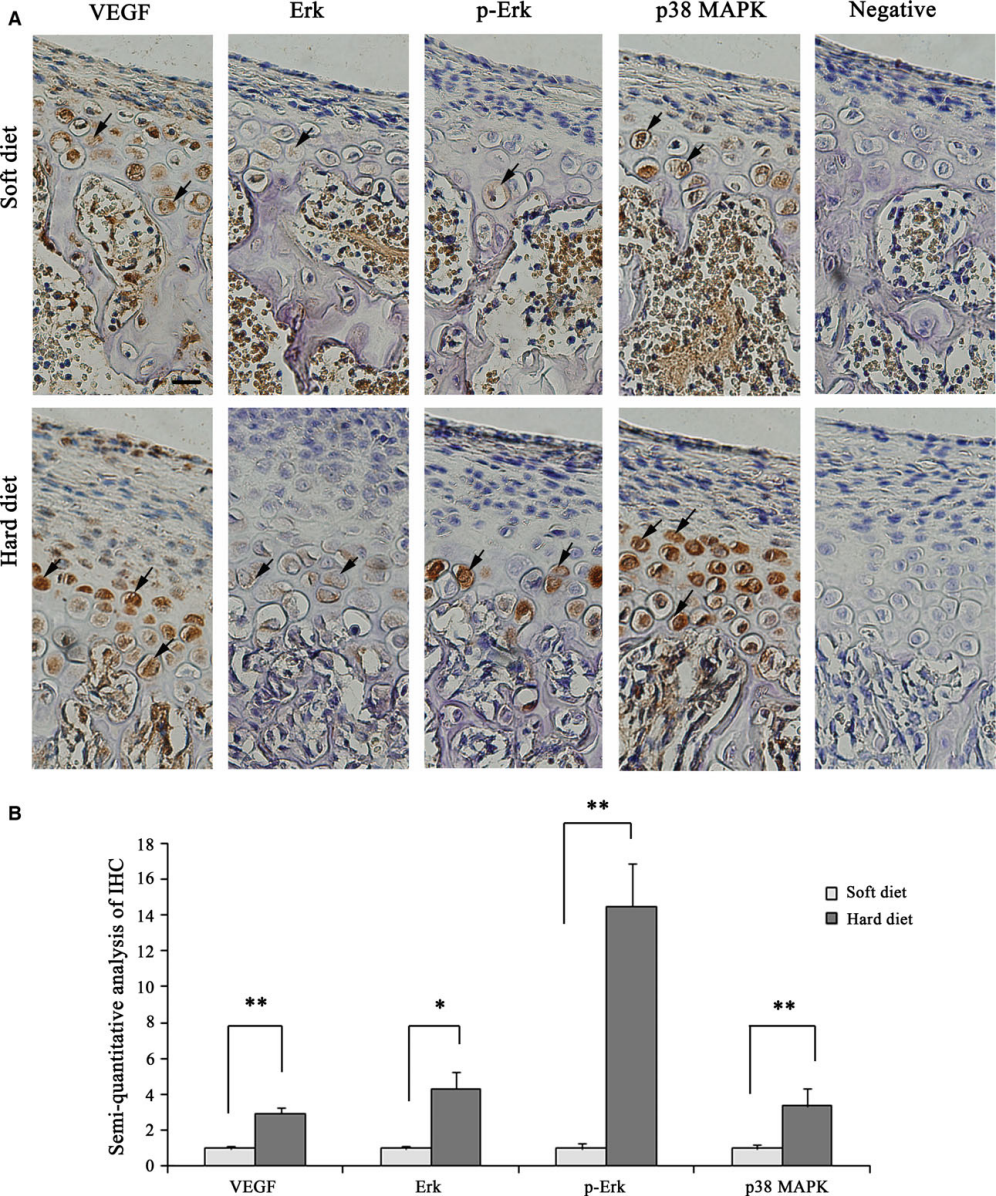
Immunostaining for VEGF, p38 MAPK and p44/42 MAPK/phospho‐p44/42 MAPK proteins (original magnification 40×) and negative controls in the anterior region of condylar cartilage in the soft and hard diet group. Bars = 20 μm. IHC staining‐positive cells were indicated by the arrows. (B) Semiquantitative analysis of VEGF, p38 MAPK and p44/42 MAPK/phospho‐p44/42 MAPK‐positive area in the soft and hard diet group (Bar graph represents the mean ± SE of three independent experiments, *P < 0.05,**P < 0.01, *t*‐test).

In the soft diet group, VEGF staining is mainly located in the proliferating and maturing layer but is very light in the hypertrophic layer (Fig. 3A). In contrast, in the hard diet group, VEGF expression was primarily observed in the hypertrophic layer. In the hard diet group, p38 MAPK‐positive and p44/42 MAPK/phospho‐p44/42 MAPK‐positive reactions were mostly detected in the maturing and hypertrophic layer in this period, while they were weak in the soft diet (Fig. 3A). Statistical analysis showed there was significantly higher VEGF, p38 MAPK and p44/42 MAPK/phospho‐p44/42 MAPK protein values in the hard diet group compared to the soft‐diet animals (Fig. 3B).

### Validation of alterations in VEGF, p38 MAPK and p44/42 MAPK/phospho‐p44/42 MAPK proteins by western blot

**Figure 4:**
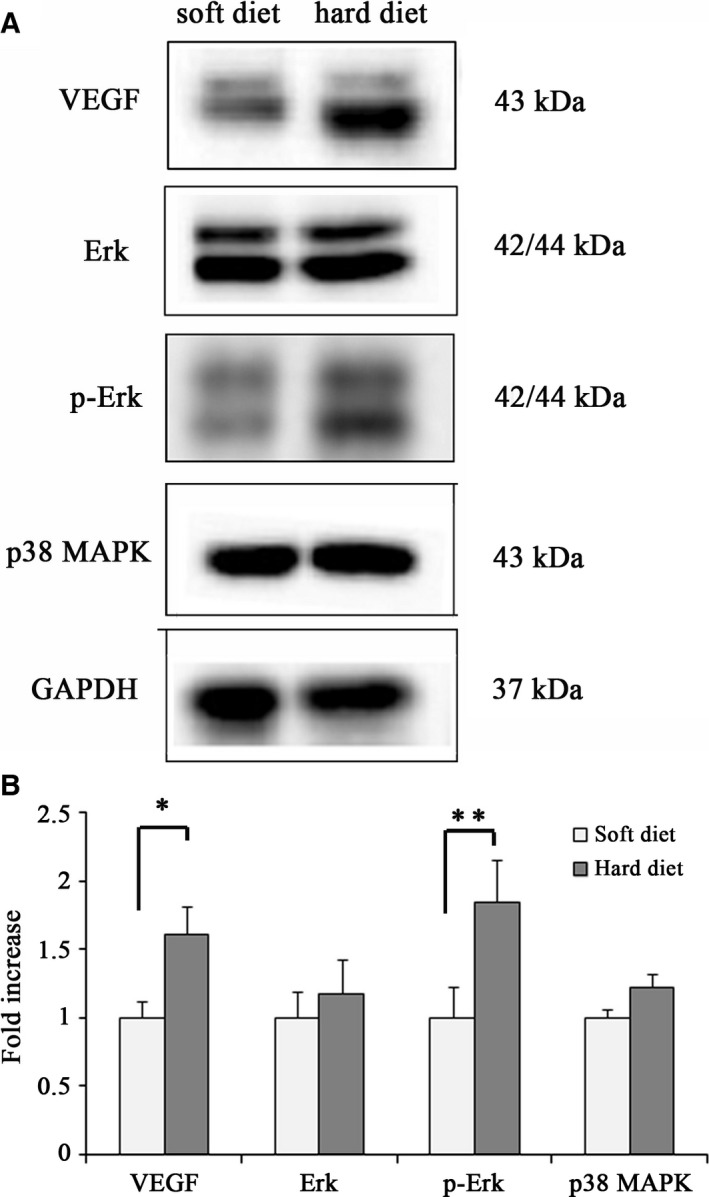
(A) Western blot of VEGF, p38 MAPK and p44/42 MAPK/phospho‐p44/42 MAPK proteins from condylar cartilage in the soft and hard diet group. Values were normalized to GAPDH. (B) Quantitation of relative protein expression (Bar graph represents the mean ± SE of three independent experiments, *P < 0.05, *t*‐test).

Four VEGF signalling‐related proteins were chosen for further identification and analysed by western blot (Fig. 4). As shown in Fig. 4B, the results revealed that VEGF, p38 MAPK and p44/42 MAPK/phospho‐p44/42 MAPK protein expression increased in the hard diet group compared to the soft diet group. The results were in agreement with current IHC and iTRAQ data, which support the proteomic analyses based on iTRAQ.

## Discussion

The normal growth and remodelling of MCC is dependent on mechanical loading and various biochemical factors 20. Recently, it was found that there was a close relationship between masticatory muscle function and condylar growth 5. Jaw muscle activity and masticatory forces of animals fed a hard diet were significantly higher than those in the soft/powdery diet animals; therefore, sufficient loading is important in maintaining the appropriate proliferation of chondrocytes and matrix production in the condyle 21. Many studies have been performed to examine the mechanism behind condylar mechanobiology. However, its signal transduction mechanisms still remain obscure and elusive 22. It has been demonstrated that the condylar cartilage undergoes endochondral bone formation, known as the replacement of cartilage with bone tissues 23. The expressions of Sox‐9, fibroblast growth factors (FGFs), transforming growth factor‐β and VEGF were found to be affected after changes in the consistency of the diet 8, 24. VEGF was initially reported to be found in the proliferative and maturing layers in the sheep condyle 25. VEGF has been implicated in endochondral ossification, angiogenesis, apoptosis of hypertrophic chondrocytes, and remodelling of extracellular matrix in both physiological and pathological conditions 20. However, little is known about the signal transduction mechanisms of VEGF under different mechanical stimulation of the condylar cartilage. Our preliminary study showed that: (a) iTRAQ‐based proteomic technology, which is a widely accepted quantitative proteomic method 26, could efficiently detect differentially expressed proteins in the condylar cartilage due to reduced masticatory function. Among the identified proteins, we found 10 VEGF signalling‐related proteins through GO analysis, KEGG pathway and PANTHER pathway analysis, which demonstrated that low‐level mechanical forces decreased the expression of VEGF *via* the MAPK signalling pathway. (b) According to the proteomic results, we further validated the presence of VEGF, p38 MAPK and p44/42 MAPK/phospho‐p44/42 MAPK proteins in the growing rat MCC and the activation of these proteins under high‐level mechanical loading conditions.

Vascular endothelial growth factor is an angiogenic factor and is considered to be mechanosensitive either in long bone 27, 28 or in condylar cartilage. According to the previous and current results, we can substantiate the following notions. First, VEGF is a good candidate for normal condylar cartilage maturation, extracellular matrix remodelling and vascular invasion, including the apoptosis of hypertrophic chondrocytes, vascular invasion and the recruitment of osteoblast progenitors. In agreement with prior literature 24, we found that in the hard diet group, VEGF expression continued in the maturing layer and hypertrophic chondrocytes. The hypertrophic layer is required for the replacement of cartilage with trabecular bone followed by vascular invasion. Yee *et al*. 25 found that VEGF was expressed mainly in the proliferative and maturing layers (early hypertrophic zone) in 18‐month‐old sheep MCC. Aoyama *et al*. 11 further reported that VEGF could not be found in new‐born MCC, and later, hypertrophic chondrocytes were positively stained for both VEGF and its receptor Flt‐1 in young rat condyles and identify the presence of VEGF, Flt‐1 and Flk‐1 proteins by western blotting, which changed with age. Furthermore, in the current study, VEGF expression increased in the hard diet group. However, abnormal mechanical stress or overexpressed VEGF may also activate the angiogenic process in adults 29 and increase the catabolic activity of chondrocytes, which could be related to the pathogenesis of osteoarthritis 14, 15, 30. These findings suggested that VEGF signalling, detected in the condylar cartilage, might stimulate the proliferation and differentiation of chondrocytes and be released into the adjacent extracellular matrix through autocrine signalling, subsequently recruit osteoblasts and chondroclasts, activate new blood vessel invasion into the hypertrophic layer and thus trigger endochondral ossification 29, 31.

Second, we hypothesized that mechanical stress induces VEGF expression *via* the p44/42 MAPK and p38 MAPK signalling pathway in condylar cartilage (Fig. 1B). VEGF binds to the VEGF receptor (VEGFR) and induces biological function. Here, we identified VEGFR2 (known as flk‐1 32) by proteomic analysis, which was detected in condylar cartilage by a previous study 11, 14. The expression of VEGFR1 in the condyle was also evident in previous studies 11. Further investigations on the regulation of VEGFRs are required. Using KEGG and PANTHER pathway analysis, we identified a series of significantly changed proteins in VEGF signalling, such as PLCG2, PLCG1, RASN, KPKB, ARAF, JUN and M3K12, which demonstrated the involvement of the MEK/ERK and p38 MAPK signalling pathways. Several studies have shown that the activation or deactivation of p38 and ERK1/2 was involved in cartilage formation and the induction of hypertrophic changes in articular chondrocytes 33. The MAPK‐AP‐1 axis (such as Fos‐ and Jun‐related transcription factors) is involved in the mechanotransduction cascade in the condyle 18. Furthermore, p38 and ERK1/2 may crosstalk with each other 33. In the present study, we validated the proteomic results by IHC and western blot and found that animals fed a powdery diet showed lower expression of VEGF, p38 MAPK and p44/42 MAPK/phospho‐p44/42 MAPK proteins in the condylar cartilage compared to the hard diet group. Otherwise, the expression and distribution of VEGF were consistent with those of the p38 MAPK and p44/42 MAPK/phospho‐p44/42 MAPK proteins. Similarly, Papachristou *et al*. 22 reported that functional alterations in the mechanical loading of condylar cartilage activated the JNK‐c‐Jun signalling pathway components and ERK/MAPK in the condylar subchondral bone, which was well correlated with the *in vitro* situation. VEGF production in response to growth factors through p38 MAPK and p44/42 MAPK were also involved in osteoblasts or in cartilage metabolism 34. It is critical to understand the mechanism by which VEGF is expressed in the condyle, which will provide the basis for future gene therapy. In addition, there might be a correlation between VEGF and other signalling proteins; therefore, further investigation should be carried out *in vivo* and *in vitro*.

In conclusion, we found that VEGF might play an important role in TMJ development and remodelling induced by alterations in functional loading through the activation of the p44/42 MAPK and p38 MAPK signalling pathway. This study provided new clues to understanding the signalling mechanism responsible for VEGF production in response to different masticatory functions at the protein level.

## Author contribution

LJ participated in the design of the study, and drafted the manuscript. YX carried out proteomic analysis. QZ carried out IHC experiments. LW carried out western blot and real‐time PCR experiments, and XS analysed the data. XJ and YG designed the project. All authors have read and approved the final manuscript.

## Supporting information


**Table S1.** List of VEGF‐related proteins by iTRAQ proteomic analysis from the condylar cartilage of rats fed 4 weeks of soft and hard food diet.
