# Supplementary material for: Identification of the vascular endothelial growth factor signalling pathway by quantitative proteomic analysis of rat condylar cartilage
Source: FEBS Open Bio. 2016 Dec 20;7(1):44–53. doi: 10.1002/2211-5463.12155 (PMC5221432; doi:10.1002/2211-5463.12155)
Supplement: Supplementary file 1 — Table S1. List of VEGF‐related proteins by iTRAQ proteomic analysis from the condylar cartilage of rats fed 4 weeks of soft and hard food diet. [file FEB4-7-44-s001.doc]

Table S1 List of VEGF -related proteins by iTRAQ proteomic analysis from the condylar cartilage of rats fed 4-weeks of soft and hard food diet.

| Accession No. | Protein name | main biological process | MW(kDa) | pI | iTRAQ quantification | |
| --- | --- | --- | --- | --- | --- | --- |
|  |  |  |  |  | 116.1/114.1 | 117.1/114.1 |
| KPCB_RAT | Protein kinase C beta type (EC 2.7.11.13) (PKC-beta) (PKC-B) | GO:0006468~protein amino acid phosphorylation;  GO:0006793~phosphorus metabolic | 88085.1 | 6.57 | 0.60 | 0.51 |
| ARAF_RAT | A-Raf proto-oncogene serine/threonine-protein kinase (EC 2.7.11.1) | GO:0006468~protein amino acid phosphorylation,GO:0006793~phosphorus metabolic process;  GO:0006796~phosphate metabolic process;  GO:0007242~intracellular signaling cascade;  GO:0007243~protein kinase cascade; GO:0016310~phosphorylation, | 74281.17 | 9.27 | 1.78 | 1.66 |
| RASN_RAT | GTPase NRas precursor (Transforming protein N-Ras) | GO:0006897~endocytosis,GO:0007010~cytoskeleton organization; GO:0007242~intracellular signaling cascade; GO:0007264~small GTPase mediated signal transduction; GO:0007265~Ras protein signal transduction;GO:0007568~aging; GO:0007569~cell aging; GO:0008284~positive regulation of cell proliferation; GO:0009967~positive regulation of signal transduction; GO:0010647~positive regulation of cell communication; GO:0010941~regulation of cell death,GO:0016044~membrane organization,  GO:0016192~vesicle-mediated transport; GO:0030029~actin filament-based process,  GO:0030036~actin cytoskeleton organization; GO:0035020~regulation of Rac protein signal transduction; GO:0035022~positive regulation of Rac protein signal transduction; GO:0042127~regulation of cell proliferation;  GO:0042692~muscle cell differentiation; GO:0042981~regulation of apoptosis, | 24399.78 | 5.02 | 2.39 | 2.96 |
| NOS3_RAT | Nitric-oxide synthase, endothelial (EC 1.14.13.39) (EC-NOS) (NOS type III) (NOSIII) (Endothelial NOS) (eNOS) (Constitutive NOS) (cNOS) | GO:0001101~response to acid,  GO:0001525~angiogenesis,  GO:0001542~ovulation from ovarian | 143436.7 | 6.8 | 0.40 | 0.58 |
| PLCG2_RAT | 1-phosphatidylinositol-4,5-bisphosphate phosphodiesterase gamma 2 (EC 3.1.4.11) (Phosphoinositide phospholipase C) (PLC-gamma-2) (Phospholipase C-gamma-2) (PLC-IV) | GO:0001775~cell activation; GO:0002253~activation of immune response,  GO:0002263~cell activation during immune response transduction,  GO:0006644~phospholipid metabolic process;  GO:0006874~cellular calcium ion homeostasis,  GO:0006875~cellular metal ion homeostasis;  GO:0006955~immune response,  GO:0007166~cell surface receptor linked signal transduction,GO:0007204~elevation of cytosolic calcium ion concentration,  GO:0007242~intracellular signaling cascade,  GO:0009395~phospholipid catabolic process | 168392 | 6.4 | 0.70 | 0.74 |
| VGFR2_RAT | Vascular endothelial growth factor receptor 2 precursor (EC 2.7.10.1) (VEGFR-2) (Protein-tyrosine kinase receptor flk-1) (Fetal liver kinase 1) (CD309 antigen) | GO:0000902~cell morphogenesis,  GO:0001525~angiogenesis,  GO:0001541~ovarian follicle | 169751.6 | 5.98 | 0.63 | 0.63 |
| KPCT_RAT | Protein kinase C theta type (EC 2.7.11.13) (nPKC-theta) | GO:0001666~response to hypoxia,GO:0001817~regulation of cytokine production; GO:0002684~positive | 92216.08 | 7.88 | 0.61 | 0.64 |
| NOS1_RAT | Nitric-oxide synthase, brain (EC 1.14.13.39) (NOS type I) (Neuronal NOS) (N-NOS) (nNOS) (Constitutive NOS) (NC-NOS) (BNOS) | GO:0001666~response to hypoxia,  GO:0002028~regulation of sodium ion transport;  GO:0002791~regulation of peptide secretion,  GO:0002792~negative regulation of peptide secretion,GO:0003012~muscle system process; GO:0003013~circulatory system process, | 179479.6 | 6.68 | 0.88 | 0.86 |
| PLCG1_RAT | 1-phosphatidylinositol-4,5-bisphosphate phosphodiesterase gamma 1 (EC 3.1.4.11) (Phosphoinositide phospholipase C) (PLC-gamma-1) (Phospholipase C-gamma-1) (PLC-II) (PLC-148) | GO:0000302~response to reactive oxygen species,  GO:0001701~in utero embryonic | 166034.3 | 5.67 | 0.87 | 1.01 |
| KPCZ_RAT | Protein kinase C zeta type (EC 2.7.11.13) (nPKC-zeta) | GO:0000226~microtubule cytoskeleton organization;  GO:0001932~regulation of protein amino acid phosphorylation;  GO:0001952~regulation of cell-matrix adhesion;  GO:0007166~cell surface receptor linked signal transduction;  GO:0007167~enzyme linked receptor protein signaling pathway;  GO:0007169~transmembrane receptor protein tyrosine kinase signaling pathway,  GO:0007242~intracellular signaling cascade, GO:0010941~regulation of cell death; | 75039.02 | 5.47 | 0.57 | 0.77 |
| VEGFD_RAT | Vascular endothelial growth factor D precursor (VEGF-D) (c-fos-induced growth factor) (FIGF) | GO:0000226~microtubule cytoskeleton organization,  GO:0001932~regulation of protein amino acid phosphorylation,  GO:0001933~negative regulation of protein amino acid phosphorylation,  GO:0001952~regulation of cell-matrix adhesion,GO:0001954~positive regulation of cell-matrix adhesion,  GO:0006461~protein complex assembly,  GO:0006468~protein amino acid phosphorylation,  GO:0006793~phosphorus metabolic process,  GO:0006796~phosphate metabolic process,  GO:0006873~cellular ion homeostasis,  GO:0007010~cytoskeleton organization | 40834.30859 | 5.91 | 0.44 | 0.54 |
| M3K12_RAT | Mitogen-activated protein kinase kinase kinase 12 (EC 2.7.11.25) (Mixed lineage kinase) (Leucine-zipper protein kinase) (ZPK) (Dual leucine zipper bearing kinase) (DLK) (MAPK-upstream kinase) (MUK) | GO:0006325~chromatin organization,  GO:0006468~protein amino acid phosphorylation,  GO:0006793~phosphorus metabolic process,  GO:0006796~phosphate metabolic process,GO:0016310~phosphorylation  GO:0016570~histone modification,  GO:0016572~histone phosphorylation,  GO:0018105~peptidyl-serine phosphorylation, | 104028.8438 | 5.81 | 0.67 | 0.76 |
| JUN_RAT | Transcription factor AP-1 (Activator protein 1) (AP1) (Proto-oncogene c-jun) (V-jun avian sarcoma virus 17 oncogene homolog) | GO:0000060~protein import into nucleus, translocation,  GO:0000302~response to reactive oxygen | 39580.91016 | 8.89 | 1.24 | 1.15 |
